# A Generative Statistical Algorithm for Automatic Detection of Complex Postures

**DOI:** 10.1371/journal.pcbi.1004517

**Published:** 2015-10-06

**Authors:** Stanislav Nagy, Marc Goessling, Yali Amit, David Biron

**Affiliations:** 1 The Institute for Biophysical Dynamics, The University of Chicago, Chicago, Illinois, United States of America; 2 Department of Statistics, The University of Chicago, Chicago, Illinois, United States of America; 3 Department of Computer Science, The University of Chicago, Chicago, Illinois, United States of America; 4 Department of Physics and the James Franck Institute, The University of Chicago, Chicago, Illinois, United States of America; Imperial College London, UNITED KINGDOM

## Abstract

This paper presents a method for automated detection of complex (non-self-avoiding) postures of the nematode *Caenorhabditis elegans* and its application to analyses of locomotion defects. Our approach is based on progressively detailed statistical models that enable detection of the head and the body even in cases of severe coilers, where data from traditional trackers is limited. We restrict the input available to the algorithm to a single digitized frame, such that manual initialization is not required and the detection problem becomes embarrassingly parallel. Consequently, the proposed algorithm does not propagate detection errors and naturally integrates in a “big data” workflow used for large-scale analyses. Using this framework, we analyzed the dynamics of postures and locomotion of wild-type animals and mutants that exhibit severe coiling phenotypes. Our approach can readily be extended to additional automated tracking tasks such as tracking pairs of animals (e.g., for mating assays) or different species.

## Author Summary

The roundworm *Caenorhabditis elegans* is a widely used model organism. Its locomotion, for instance, enables the study of genetic and cellular mechanisms that underlie behavior and may be broadly conserved. Characterizing *C*. *elegans* locomotion requires identifying its body posture and tracking how posture changes with time. Existing machine vision approaches have greatly aided this effort. However, they have been limited in cases where the body of the animal curved strongly such that one part of the animal rested or slid against another part. We present a method for automated detection of such complex body postures and its application to the analysis of locomotion. At the core of our method are progressively detailed statistical models of the shape of the animal. These models enable us to assess the probability that a given image contains a suggested posture. Our approach does not require manual initialization and can be readily parallelized for large-scale applications. We used our approach to analyze locomotion in mutants that severely exaggerate their body bends, called coilers. This approach can readily be extended to additional automated tracking tasks such as pairs of interacting roundworms or different organisms.

This is a *PLOS Computational Biology* Methods article

## Introduction

The nematode *Caenorhabditis elegans* is a simple animal model system, widely used to study the genetic foundations of behavior. Among its key advantages are its tractable genetics, short life cycle, relatively simple anatomy and behavior patterns, and evolutionary conserved pathways [1–3]. The locomotion patterns of *C*. *elegans* have been extensively studied. Historically, this was largely done relying on visual phenotyping.

In recent years, several machine vision tools have been developed for automated posture analysis, collectively referred to as “trackers”, and spanning a range of capabilities [4–10]. Accurate identification of head and tail and reconstruction of the midline of the body are important steps in automated analyses of *C*. *elegans* postures. Typically, the topological genus of images of wild-type animals is zero, i.e., the body image only rarely forms closed loops. However, loops are observed in coiling mutants and more rarely in wild-type or other mutants.

Existing trackers were rarely used to automate the study of severe coiler phenotypes, plausibly because in such cases they either require frequent manual intervention or may misidentify the posture [11–14]. Therefore, we refer to such non-self-avoiding postures as complex.

Generative statistical models describe the expected images given a particular posture. This expectation is formulated in terms of a probability distribution, referred to as the data or likelihood term [15]. Knowledge about the data, such as expected body length or smoothness, is accounted for by specifying an a priori distribution of postures. The algorithm then optimizes the posterior probability of the posture, i.e., the product of the likelihood and the prior term. This framework can enable the identification of complex postures.

Here, we present a posture detection method based on generative statistical models and a coarse-to-fine strategy. Our approach allows a computationally efficient implementation and yields reliable detections of many complex postures. First, a small set of characteristic features for the head and body regions is defined as functions of oriented edges in the image. Next, we formulate a statistical model describing the likely configurations of these features given a hypothesized posture. At run time, we search for the maximum a posteriori posture of the worm given the observed image. This calculation yields a coarse outline of the animal.

To refine the outline, a second statistical model is employed which directly uses the edge information in the image and hence enables more precise identification. The advantage of this coarse-to-fine technique is computational efficiency. The coarse search runs over a grid which is much smaller than the original image grid. The fine search is then initialized using the result of the coarse detection and is only required to explore a small subset of possibilities. Searching for the posture at the fine scale without considering the information obtained from a coarse search would have been computationally intractable. To demonstrate the utility of our method, we assayed wild-type animals and several mutant strains that were previously associated with a coiler phenotype.

## Results

### A coarse statistical model can efficiently approximate complex postures

A coarse statistical model was defined to identify approximate positions of the head, tail and midline of the animal. The model is based on prominent features of the head and the body that are identified on a coarse-grained grid *H*, in which every point corresponds to a block of pixels in the original image grid *G*. Through trial and error, we found that a coarse grid unit length of one quarter of the width of the worm offered a good tradeoff between efficiency and accuracy. The key components of this model and the resulting detection are descried below.

#### (i) Body and head features

**Fig 1:**
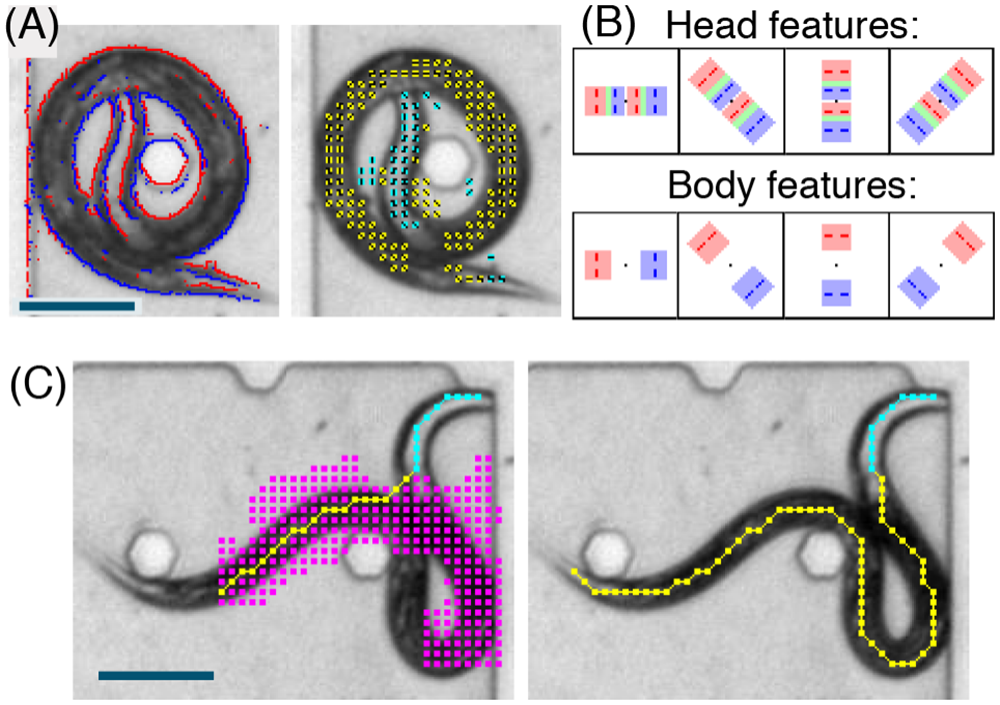
(A) Left: detected edges in an image of a wild-type animal exhibiting a (rare) coiled posture. The red/blue colors indicate the two possible edge polarities. Right: Detected body (yellow) and head (cyan) features with indicators of the corresponding orientations. (B) Graphical representations of the four head features and the four body features. Each feature corresponds to a spatial arrangement of areas with elevated probabilities for specific edge types. The red and blue shade colors represent areas with increased probabilities for positive and negative polarity edges, respectively. Green areas represent an overlap of red and blue areas. Red and blue dashed lines represent the orientation of positive and negative polarity edges, respectively. These masks introduce invariance to the exact location of the edges and are hence robust to variations in the width of the worm. (C) Left: a detected subinstantiation at an intermediate search step. The magenta blocks indicate which coarse locations have been visited so far during the search for the body. Right: Final detection of the posture using the coarse detection algorithm. Scale bars represent 100 μm.

To construct mid-level local image features which are characteristic for the body or head region of the worm, we follow the approach in [15] and use a set *E* of eight types of binary edges corresponding to four possible orientations (horizontal, vertical, and diagonal) with two possible polarities. Edges in the digitized image are detected by computing the intensity difference between adjacent pixels. If this difference exceeds the edge detection threshold (S1 Table) and the magnitude of the difference exceeds the magnitude of the differences between neighboring pairs, then the presence of the corresponding edge is noted and its polarity is determined by the sign of the difference (Fig 1A). Multiple edge orientations can be detected at the same location. This yields edge arrays *X*_*e*_ for *e* ∈ *E*, where *X*_*e*_(*y*) = 1 if an edge of type *e* was found at pixel *y* ∈ *G* and *X*_*e*_(*y*) = 0 otherwise (Fig 1A). The binary criteria for identifying edges are largely photometric invariant, i.e., insensitive to details of brightness and contrast. This makes our method robust to changes in imaging conditions (see, e.g., S1 Fig).

The edge configurations in body and head regions of the worm can be described through simple masks (Fig 1B). Body segments exhibit inversely polarized edges separated by a characteristic distance and head segments exhibit inversely polarized outer edges as well as inversely polarized inner edges. We use one mask for each of four head segment orientations and one mask for each of four body segment orientations. The size of these feature masks is scaled to the expected worm width. Formally, the image features are defined as statistical models for local regions of the edge arrays with elevated probabilities for corresponding edges in the specified areas. It is assumed that edges are independent given the feature type. Feature detection is based on likelihood ratio tests, which compare each of these models to a background model under which edges of all types have a low probability. The null hypothesis that the edges follow the background model is rejected whenever the number of corresponding edges in the specified areas exceeds a threshold (S1 Table). The oriented feature is then declared to be `on’ at the test location and registered on the coarse grid *H* (Fig 1A). This results in feature arrays *X*_*f*_(*Z*) for *f* ∈ *F*,*z* ∈ *H*, where *F* is the set of eight features types.

#### (ii) Admissible instantiations and the prior

The posture of *C*. *elegans* in a given image is described by a sequence $\vec{\theta }=(\theta_{1},\ldots ,\theta_{n})$ of neighboring points on the coarse grid *H*. The locations of these points as well as the length of the sequence are unknown. Biomechanical constraints limit the maximal curvature that the body of the animal can support [7]. We thus restrict the set of admissible instantiations to incorporate the smoothness of attainable postures into our model. We require that for each triple of points *θ*_*i*-1_,*θ*_*i*_,*θ*_*i*+1_ the directions defined by {*θ*_*i*-1_,*θ*_*i*_} and {*θ*_*i*_,*θ*_*i*+1_} differ by either *0°* or *±45°*. In other words, given *θ*_*i*-1_ and *θ*_*i*_ there are three possible positions for *θ*_*i*+1_. An additional hard constraint we use is that the midline (but not necessarily the entire body) of the animal is self-avoiding, i.e., every point can appear at most once in the sequence. This constraint can be relaxed and replaced by a penalty term for self-crossing. Taken together, these restrictions successfully suppress erroneous detections corresponding to postures that are not naturally observed while maintaining sufficient flexibility for approximating all observed postures.

A reliable coarse detection of the posture can typically be obtained using a uniform prior on the space of admissible instantiations. However, we found that our method identifies complex postures more robustly when information about the expected length of the animal was taken into consideration. This was done using the prior *P*(*θ*) exp[−*A*(*n*−*λ*)^2^], where *n* is the number of points in the sequence, *λ* is the expected length of the worm and A > 0 is a constant that determines the precision of the prior.

#### (iii) The data model

We now specify the probability distribution of the features given an instantiation $\vec{\theta }$. As seen in Fig 1A, features with the correct orientation appear on the midline with high probability and in the vicinity of the midline with medium probability. Features with different orientations, as well as any features away from the midline, occur with low probability. This motivated the following model.

We denote the feature type with orientation parallel to the direction {*θ*_*i*-1_,*θ*_*i*_} by *f*_*i*_. For each point *θ*_*i*_ in the sequence $\vec{\theta }$ we denote the two nearest neighbors in the direction orthogonal to {*θ*_*i*-1_,*θ*_*i*_} by *θ*_*i*−_ and *θ*_*i*+_. In addition, define *H*_*θ*_ to be the set of locations on the coarse grid consisting of all points in $\vec{\theta }$ and all of their respective orthogonal neighbors. Assuming that all features are conditionally independent given $\vec{\theta }$ we obtain:

$$\forall i\;P\left(X_{f}(\theta_{i}),f\in F|\theta \right)=p_{high}{}^{X_{f_{i}}\left(\theta_{i}\right)}{\left(1-p_{{}_{high}}\right)}^{1-X_{f_{i}}\left(\theta_{i}\right)}\cdot \prod_{f\neq f_{i}}p_{low}{}^{X_{f}\left(\theta_{i}\right)}{\left(1-p_{{}_{low}}\right)}^{1-X_{f}\left(\theta_{i}\right)}$$ (1)

$$\forall i\;P\left(X_{f}(\theta_{i\pm }),f\in F|\theta \right)=p_{med}{}^{X_{f_{i}}\left(\theta_{i\pm }\right)}{\left(1-p_{med}\right)}^{1-X_{f_{i}}\left(\theta_{i\pm }\right)}\cdot \prod_{f\neq f_{i}}p_{low}{}^{X_{f}\left(\theta_{i\pm }\right)}{\left(1-p_{{}_{low}}\right)}^{1-X_{f}\left(\theta_{i\pm }\right)}$$ (2)

$$\forall y\notin H_{\theta }\;P\left(X_{f}(y),f\in F|\theta \right)=\prod_{f\in F}p_{low}{}^{X_{f}\left(y\right)}{\left(1-p_{{}_{low}}\right)}^{1-X_{f}\left(y\right)}$$ (3)

Estimates for *p*_*high*_, *p*_*med*_, and *p*_*low*_ were obtained from regions in a small number of images where body and head segments were identified manually together with their orientation. With this data model the posterior probability of an instantiations $\vec{\theta }$ will be high if the orientations {*θ*_*i*-1_,*θ*_*i*_} are in agreement with the detected features.

#### (iv) The coarse detection algorithm

The posture detection algorithm starts by exclusively using the head features in order to identify a sub-instantiation (*θ*_1_,…,*θ*_*m*_) corresponding to the head of the worm. This leverages the observation that head features are much sparser than body features. Also, simple heuristics can be used to find candidates of starting points (*θ*_1_,*θ*_2_). After the head is identified a search starting from either end (*θ*_1_ or *θ*_*m*_) is performed to detect the body and determine the full instantiation (*θ*_1_,…,*θ*_*n*_).

The algorithm summarized below successively searches for instantiations that possess one additional point while keeping track of multiple instantiations as long as they have potential to grow to the instantiation with overall best posterior.

Input: feature maps *X*_*f*_, feature probabilities *p*_*low/high/medium*_, a priori length *λ* and prior precision *A*.

1. Identify the starting points (*θ*_1_,*θ*_2_) and initialize the variables *candidates*←{(*θ*_1_,*θ*_2_)} and *best*←{(*θ*_1_,*θ*_2_)}.
2. For *n = 3*, *4*,…
  - Find admissible instantiations {*inst*_1_,*inst*_2_,…} of length n by adding a single point to each of the instantiations from *candidates* (there are at most three possible extensions for each candidate due to the constraint on triplets of consecutive points). Empty the list *candidates*.
  - Calculate the posterior of the new instantiations {*inst*_1_,*inst*_2_,…}.
  - If log[*posterior*(*inst*_*n*_)] ≥ log[*posterior*(*best*)]−*DIFF* store the instantiation *inst*_*n*_ in *candidates* (for multiple instantiations with the same endpoint only store the best one).
  - Replace *best* if the instantiation with the highest posterior is better.
3. Stop the loop when no improvement occurred, i.e., when *best* was not replaced for a small number of iterations (2 for the head search and 3 for the body search).

Output: detected coarse instantiation (*θ*_1_,…,*θ*_*n*_).

It is important to keep searching for a few iterations even if no improvement was found in order to be able to pass through regions where features are missing. The parameter *DIFF* determines how permissive the search is, i.e., how many candidates are tracked at a time. Throughout this work, we used *DIFF* = *α*{[log(*p*_*high*_)+2log(*p*_*med*_)−3log(*p*_*low*_)]−[log(1−*p*_*high*_)+2log(1−*p*_*med*_)−3log(1−*p*_*low*_)]} with *α* = 3. In words, this is a small multiple of the difference in log-likelihood ratio between a perfect and a worse case additional point for a coarse instantiation.

Our algorithm is adaptive in the sense that multiple hypothetical body midlines are traced until the uncertainty is resolved. In regions with informative features the procedure essentially behaves as a depth-first search. However, in regions with no detected features it works as a breadth-first search. Fig 1C shows how the detected posture can change as the search progresses and more data are incorporated into the evaluation. In our experiments, the candidate list typically consisted of 10 to 30 instantiations and the runtime of the coarse detection was approximately 100 ms on a standard desktop machine, where approximately half of the time was spent to compute the edges and the features.

### A fine statistical model can accurately identify complex postures

The estimated point sequence $\vec{\theta }$ on the coarse grid facilitates finding the boundaries of the animal body at the resolution of the original grid, from which a refined midline can be derived. The key components of this model and the resulting detection are described below.

#### (i) Admissible instantiations and the prior

**Fig 2:**
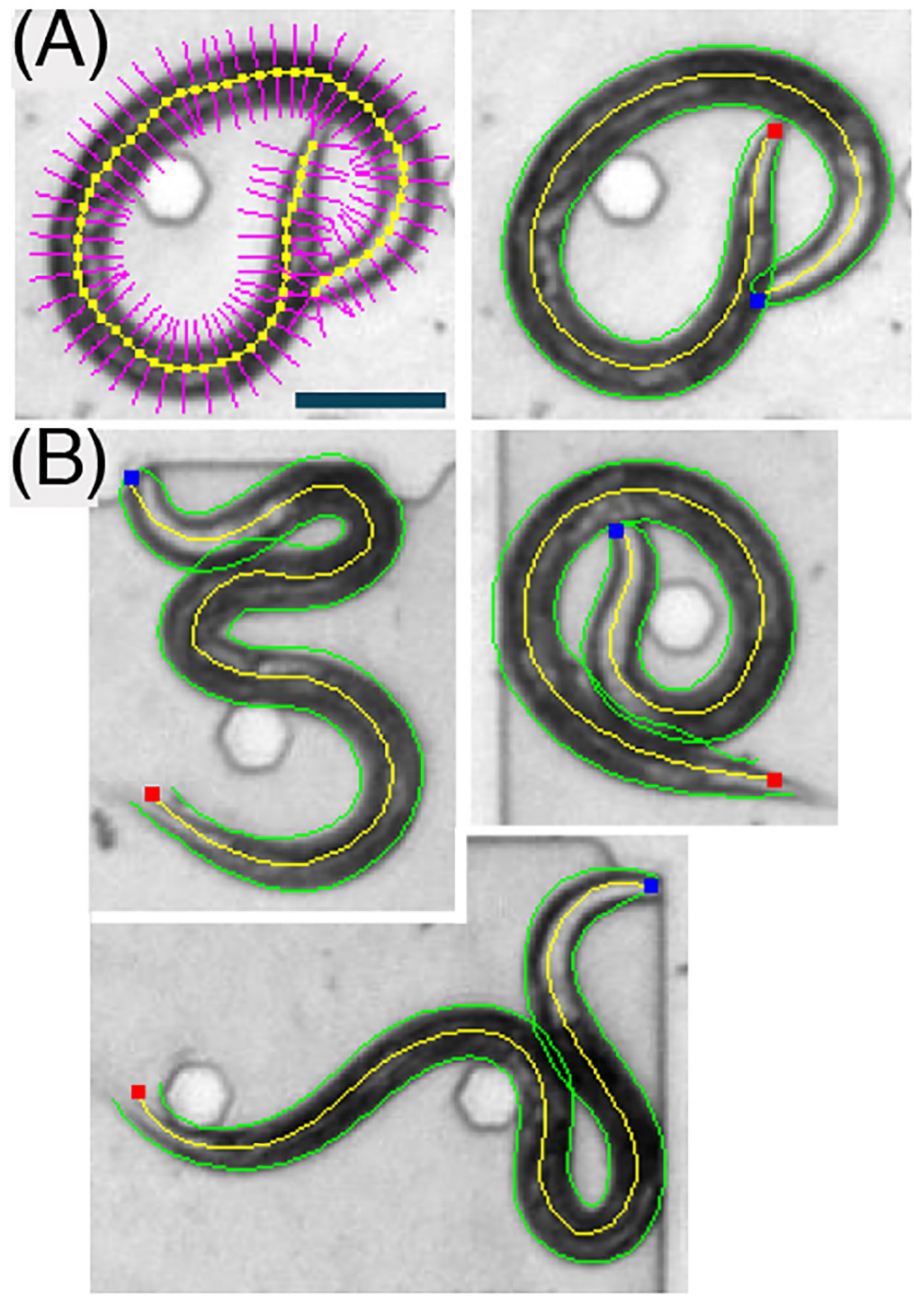
(A) Left: a smoothed midline and admissible regions (magenta) for the fine detection of the posture of a wild-type animal. Right: the corresponding fine instantiation. (B) Additional examples of detected non-self-avoiding postures, rarely exhibited by wild-type animals. The midline and edges of the animal are depicted in yellow and green, respectively. The head and the tail of the animal are depicted in blue and red, respectively. Scale bar represents 100 μm.

The boundaries of the animal body are described through a sequence of points $\vec{\eta }=\left(\eta_{0},\ldots ,\eta_{n},\ldots ,\eta_{2n}\right)$ on the image grid *G*. The points *η*_0_ and *η*_2n_ correspond to the left and right side of the tail, while *η*_*n*_ corresponds to the tip of the head. For every point *η*_*i*_ a small subset *G*_*i*_ ⊂ *G* of admissible locations is derived from the coarse instantiation (see Fig 2A). In addition, the expected orientation for each line segment {*η*_i−1_,*η*_*i*_}, denoted *β*_*i*_, is determined from the corresponding orientation of the coarse detection. The prior $P\left(\vec{\eta }\right)∼\text{exp}\left(-B\sum_{i}\left|\alpha_{i}-\beta_{i}\right|\right)$ is then imposed on the instantiations $\vec{\eta }$, where *a_i_* denotes the actual orientation of the line segment {*η*_*i*−1_,*η*_*i*_} and B > 0 is a constant that determines the precision of the prior.

#### (ii) The data model

We approximate the orientations *α*_*i*_ to be horizontal, vertical or at *±45°*. This allows us to associate each line segment {*η*_*i*−1_,*η*_*i*_} on the image grid *G* with an edge type *e*_*i*_. The statistical model for the original edge data is then motivated by the following observations. Edges with the appropriate orientation appear on the boundaries of the body with a high probability *p*_*obj*_. However, edges of different orientations at these locations or any edge away from the boundaries of the body are only detected with low probability *p*_*bg*_. Analogously to the coarse model we assume that the edges *X*_*e*_(*y*), *e* ∈ *E*, *y* ∈ *G* are conditional independent given the instantiation $\vec{\eta }$:

$$\forall y\in \left[\eta_{i-1},\eta_{i}\right]\;P\left(X_{e}(y),e\in E|\eta \right)=p_{obj}{}^{X_{e_{i}}\left(y\right)}{\left(1-p_{obj}\right)}^{1-X_{e_{i}}\left(y\right)}\cdot \prod_{e\neq e_{i}}p_{bg}{}^{X_{e}\left(y\right)}{\left(1-p_{{}_{bg}}\right)}^{1-X_{e}\left(y\right)}$$ (4)

$$\forall y\notin \text{any line segment }P\left(X_{e}(y),e\in E|\eta \right)=\prod_{e\in E}p_{bg}{}^{X_{e}\left(y\right)}{\left(1-p_{{}_{bg}}\right)}^{1-X_{e}\left(y\right)}$$ (5)

#### (iii) The fine detection algorithm

The boundaries of the body can efficiently be detected with a dynamic programming procedure, successively reducing the problem by one variable. This is possible because the log-posterior is of the form $\text{log}P\left(\vec{\eta }|\left\{X_{e}(y)\right\}\right)=\sum_{i}g_{i}\left(\eta_{i-1},\eta_{i}\right)$, i.e., it is a sum of functions of successive points in the sequence $\vec{\eta }$. A detailed presentation of dynamic programming for a similar statistical model can be found in [15]. The resulting boundaries and refined midlines for sample images are shown in Fig 2B. The runtime of the fine detection procedure is typically less than 50 ms on a standard desktop machine.

#### The statistical framework can be readily extended or refined

Although the approach as described above offered significant improvement as compared to traditional trackers, it exhibited limited success rates for certain classes of postures. These included fully and tightly coiled postures, which readily occurred when body boundaries remained in continuous contact for extended periods, or postures that were enriched when a large number of collisions of the head and the tail occurred in quick succession. In order to enable the detection of such difficult postures we designed a more sophisticated version of the algorithm for the analysis of a single image.

**Fig 3:**
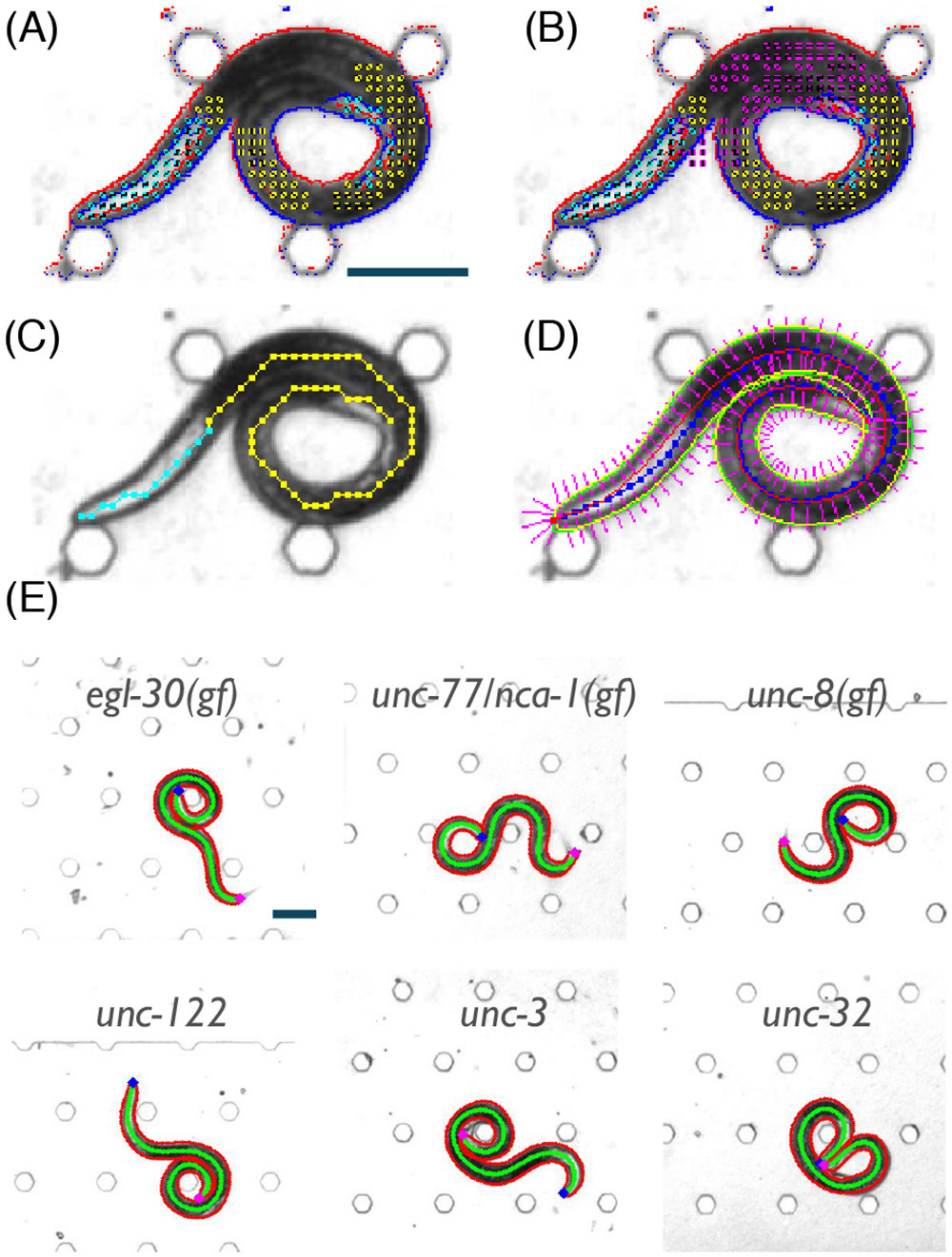
(A) Head (cyan) and body (yellow) features of an *egl-30* mutant, as determined by the basic algorithm. (B) Same as (A) with added double-body (magenta) features, as determined by the improved algorithm. (C) Coarse detection of the posture of the *egl-30* mutant using the improved algorithm. (D) Fine detection of the posture of the *egl-30* mutant. (E) Examples of non-self-avoiding postures detected in six mutants that exhibit coiler phenotypes. The midline and edges of the animal are depicted in green and red, respectively. Scale bars represent 100 μm.

First, we introduced an additional body feature where the edge areas are two worm widths apart instead of one. If the test is positive corresponding features are placed at the hypothesized centers of the two body segments. This was necessary in order to detect locations where two parts of the body aligned against each other, which previously led to regions with missing features. Second, the coarse statistical algorithm was changed to retain multiple candidate sub-instantiations for the head (one for each cluster of head features) rather than simply choosing the most likely one. Often, only a single cluster of head features was present, but in complex cases 2–3 clusters may have been generated. A search for the full head and body instantiation was then initiated using each of the candidate head detections $\left(\theta_{1}^{\left(j\right)},\ldots ,\theta_{m\left(j\right)}^{\left(j\right)}\right)$. The full instantiation $\left(\theta_{1}^{*},\ldots ,\theta_{n}^{*}\right)$ with the highest posterior was declared as the solution to the detection problem. Avoiding an all-or-none decision at the stage of the coarse head identification enabled treatment of false positive detections of head features, which were a key cause of errors in the basic implementation. An example of the resulting detection steps in a case where large portions of the body boundaries are in continuous contact is shown in Fig 3A–3D and S1 Movie. The total runtime of this improved search was typically less than 200 ms per frame on a standard desktop machine.

These modifications demonstrate the flexibility of the proposed approach: image features can be replaced or added in response to the appearance of the observed object. More generally, adapting the statistical model or the search algorithm allows for improving detection in a wide range of experimental conditions. We note that no additional parameters or tuning of the existing values was necessary. All parameter values, as listed in S1 Table, were set once during the development of the analysis code and were not further tuned for any of the experiments.

### Statistical image detection enables automatic characterization of coiling phenotypes

To test the proposed algorithm we assayed wild-type animals and mutants that were previously reported to exhibit coiling. Of particular interest were three strains with severe locomotion defects. The G_q_ protein alpha subunit ortholog, encoded by *egl-30*, was shown to affect locomotion, viability, egg laying, synaptic transmission, and pharyngeal pumping [16–28]. The voltage-insensitive cation leak channel, a subunit of which is encoded by *unc-77/nca-1*, assists transmission of presynaptic activation from the cell body to the synapses [29,30]. The *unc-8* gene encodes a putative mechanosensory channel [31]. A gain-of-function (gf) allele of either of these genes result in exaggerated body bends and coiling [27,30,31].

**Fig 4:**
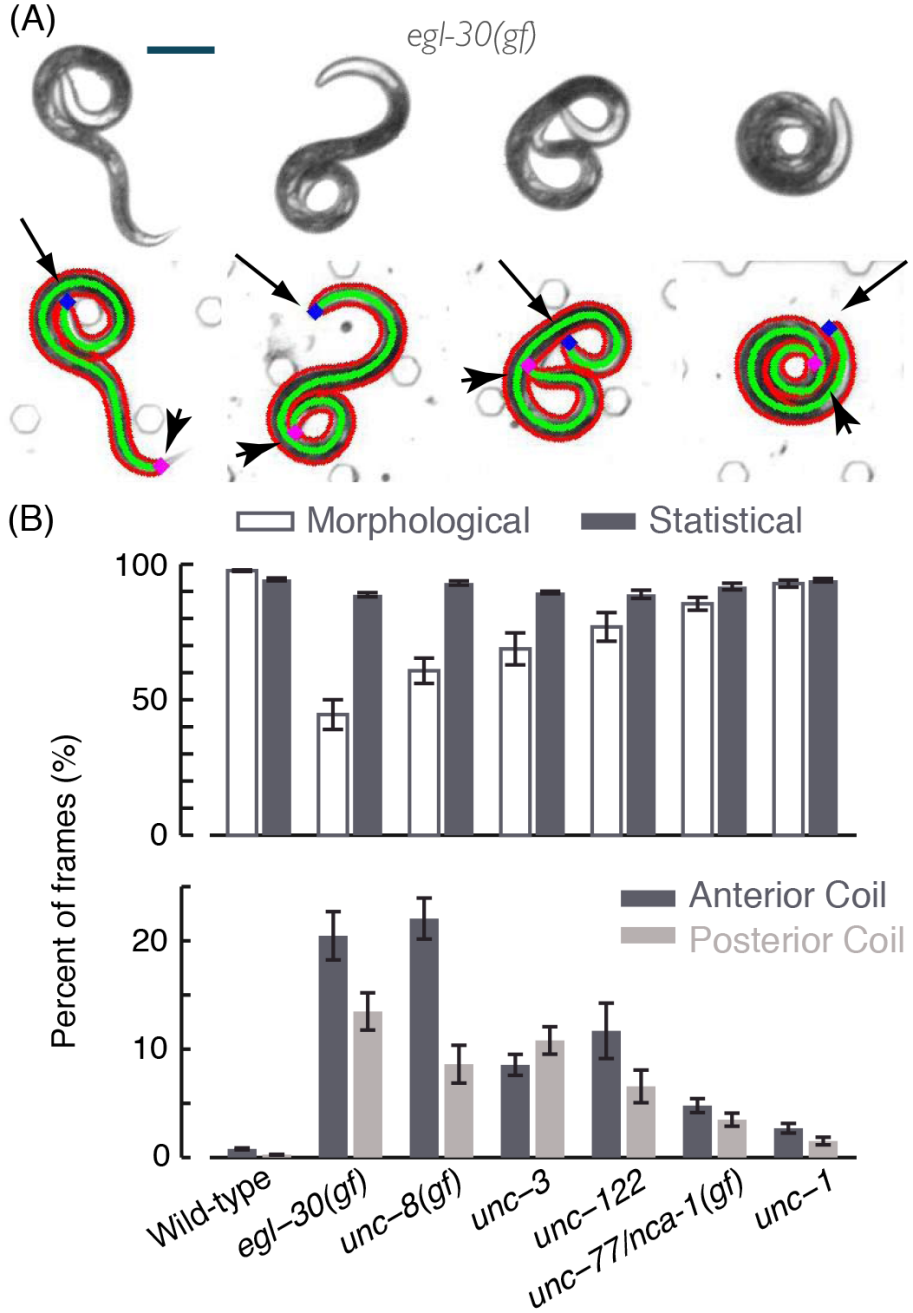
(A) Anterior coils and posterior coils exhibited by an animal carrying the *egl-30(gf)* allele. Arrows and arrow-heads point to the head (blue) and tail (magenta) of the animals, respectively. (B) Top: the percentage of frames in which posture was successfully identified in wild-type animals and coiler mutants using morphological operations or a generative statistical model. Bottom: the percentage of frames in which a coiled posture was detected. In panel (B), 9–12 L4 larvae of each genotype were imaged at 10 frames per second for 2–4 hours, yielding a total of approximately 10^5^ images. Error bars and thin lines depict animal-to-animal variation (mean ± s.e.m).

Examples of successfully detected complex postures for six mutants that display a coiler phenotype are shown in Fig 3E and S2 Movie. In this work, anterior coils were defined as periods when the head was in close proximity to any point along the body (within 5% of the midline). Posterior coils were similarly defined for the tail. We note that these definitions were not mutually exclusive (Fig 4A). The rate of detections using the generative statistical algorithm was compared to that of a previously described image analysis tool [9], which uses a standard morphological approach for single-frame detection that solely relies on the contrast between object and background (see also the Discussion section). The statistical approach yielded midlines of appropriate length for >90% of the images and these midlines were very similar to those obtained using the morphological approach, when the latter was available. For coiler mutants, the differences between the two algorithms mirrored the abundance of coils, indicating that detecting complex postures was key to the observed improvements (Fig 4B).

**Fig 5:**
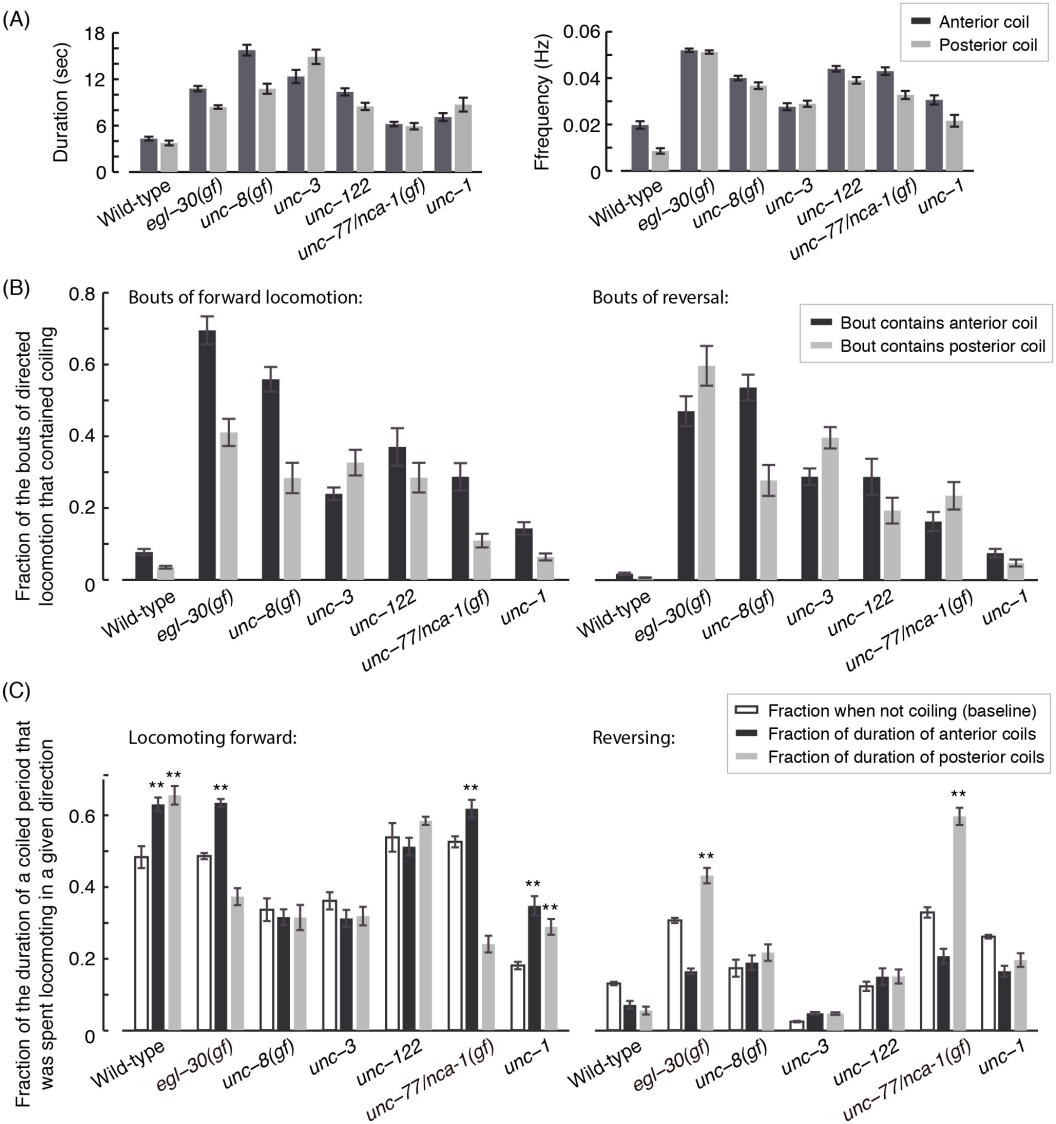
(A) The characteristic duration (left) and frequency (right) of continuous periods of coiling. (B) The fraction of bouts of forward (left) and backward (right) locomotion that contained coiled postures. The majority of bouts of directed locomotion of *egl-30(gf)* and *unc-8(gf)* mutants involved coiled postures. (C) A continuous period during which the animal was coiled, termed a coiling event, could contain sub-periods of forward or backward locomotion in addition to dwelling. Filled bars depict the fractions of the durations of coiling events that were spent exhibiting locomotion in a given direction. The mean propensities for directed locomotion when the animal was not coiling (empty bars) were measured as a baseline for comparisons. Asterisks denote that the propensity during coiling of the locomotion state in question was higher than its corresponding baseline (p<0.01). In all panels, 9–12 L4 larvae of each genotype were imaged at 10 frames per second for 2–4 hours, yielding a total of approximately 10^5^ images. Error bars and thin lines depict animal-to-animal variation (mean ± s.e.m).

As a coarse measure of the severity of different coiling defects, the durations and frequencies of coiling were measured for each of the strains tested. Typical timescales were obtained by fitting the data to a Weibull distribution [32] (Fig 5A) and the full distributions for a severe coiler, *egl-30(gf)*, are depicted in S2 Fig. The proposed algorithm thus enabled us to obtain a nearly continuous record of posture dynamics, uninterrupted by coiling.

To examine the relation between coiling and locomotion, we derived the propagation of dorsoventral body-bends from the time-series of postures as previously described (see Materials and Methods section and [9]). Coiling events in wild-type animals were rare, their durations were short, and they rarely interrupted directed locomotion (Fig 5A and 5B). In contrast, the majority of bouts of directed locomotion were interrupted by coiling in *egl-30(gf)*, and *unc-8(gf)* mutants and coils were longer and more frequent than wild-type in these mutants (Fig 5A and 5B).

During continuous periods in which the posture of *C*. *elegans* is non-self-avoiding, the directional propagation of body bends can be disrupted to varying degrees. Therefore, an alternative measure of the impact of coiling can be obtained by asking how it affects directed locomotion. To address this, we measured the propensities for directed locomotion during coiling events. In each case, these propensities were compared to their baseline values, i.e., when coiling was absent (Fig 5C). Wild-type animals mostly progressed forward during a coil. This was the case since wild-type coiling was largely caused by Ω-turns which facilitate turning and are not detrimental to directed locomotion (see below). In *egl-30(gf)* and *unc-77/nca-1(gf)* mutants, propensities for forward or backward locomotion exceeded their baseline levels during anterior or posterior coiling, respectively (Fig 5C). Thus, in these mutants directed propagation of dorsoventral bends could be sustained despite coiling and, as further shown below, locomotion and coiling were likely linked. Taken together, these results suggest that the proposed statistical approach can be used to characterize coiler phenotypes.

### Coiler phenotypes can be intuitively classified using projections to low-dimensional spaces

Principal component analysis (PCA) was proposed as an unbiased and efficient approach for describing *C*. *elegans* behavior [7]. It has been used to characterize the dimensionality and dynamics of locomotion, as well as behavioral motifs [7,8,33,34]. When complex postures are largely inaccessible, two of the leading modes describe sinuous oscillations associated with directed locomotion and a third is associated with turning [7,34].

**Fig 6:**
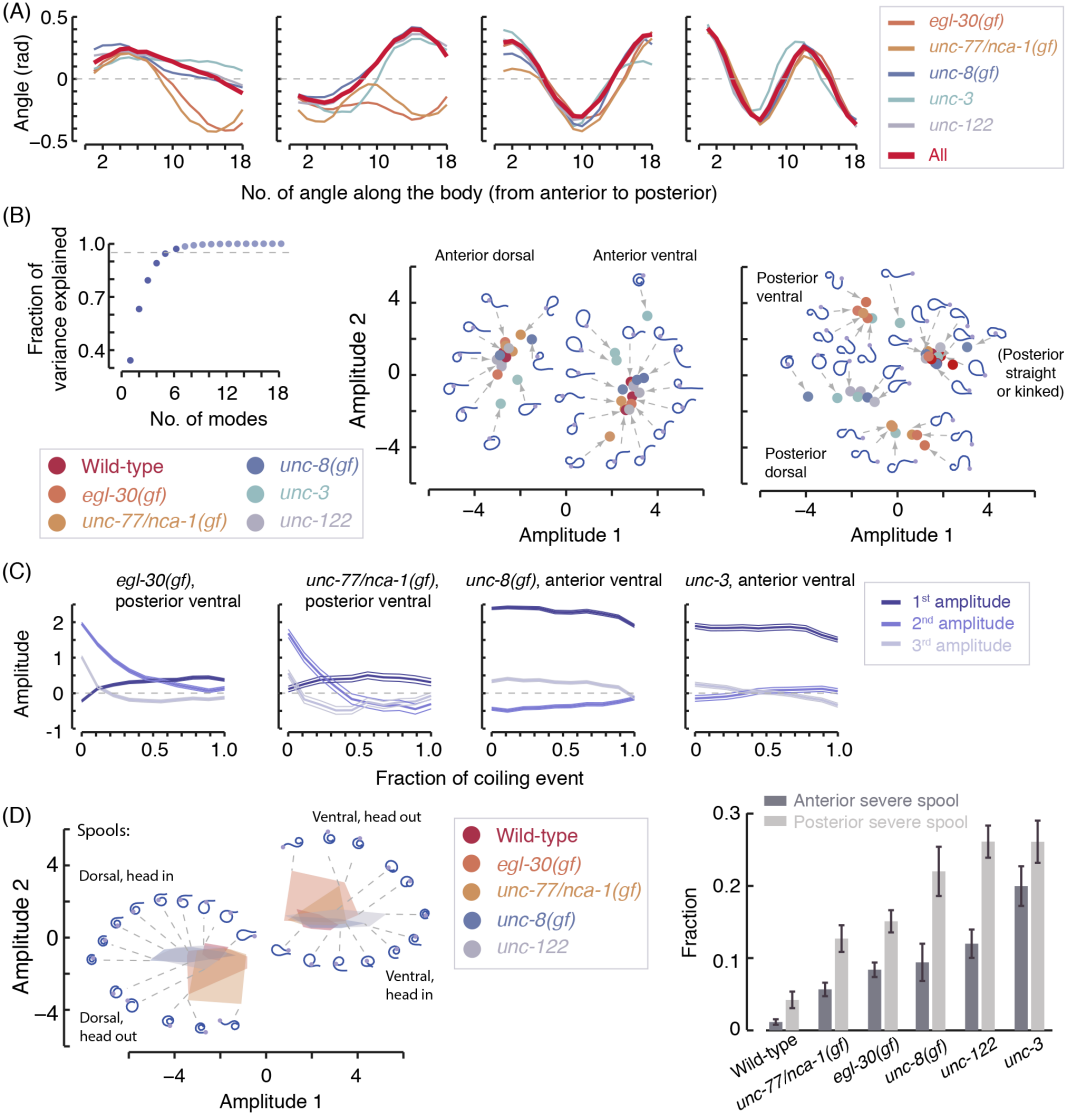
(A) The leading four PCA modes for individual coiler mutants (thin lines) and for the combined coiler dataset (thick lines). (B) Left: the variance explained by the modes of the combined coiler dataset in order of their significance. Dashed line represents 95%. Middle/right: centroids of posture clusters were projected onto the plane of the two leading modes. Typical coiling postures were reconstructed from centroids. The intuitive interpretation of the two leading modes is demonstrated by the separation between ventral (positive amplitudes) and dorsal (negative amplitudes) coiling. (C) The dynamics of the amplitudes of the three leading modes during continuous periods of coiling. The duration of individual coiling events were normalized such that the horizontal axis depicts the fraction of duration of the coiling event (mean durations for each strain can be seen in Fig 5A). (D) Left: for each strain shown, the 10 most populated cluster centroids for spools (coiled postures for which *a*_1_·*a*_2_ > 1) were projected onto the plane of the two leading modes and their convex hull was calculated. These convex hulls for wild-type animals (red), static coilers (blue shades) and loopy movers (orange shades) are shown in the plane of the two leading modes. Example postures were reconstructed from the cluster centroids (blue curves). Dotted lines point from the position of a cluster centroid to the reconstruction of its respective body posture. Grey circles at edges of reconstructed postures denote the position of the head. Right: the fraction of severe spools exhibited by wild-type animals and coiler mutants in our assays. Error bars and thin lines depict animal-to-animal variation (mean ± s.e.m). Eigenworms are represented using angle differences (49) as opposed to angles with a fixed axis (7) (see Materials and Methods).

We constructed an ensemble of complex postures by equally sampling the coiled postures of the mutants we assayed. The resulting two leading modes were associated predominantly with anterior and posterior curvature. Thus, the severity and direction (dorsal/ventral) of anterior and posterior coils corresponded to the amplitudes and signs of these modes, respectively (Fig 6A and 6B). The third mode contributed opposite curvatures to the edges and the mid-body. Additional modes introduce higher order corrections and more than 95% of the variance in the data was accounted for by the leading six modes (Fig 6A and 6B).

Identifying typical coiling postures is ambiguous due to their broad distributions. Nevertheless, a heuristic definition can highlight prominent features and provide a useful starting point. We used k-means clustering to sub-divide the dataset of amplitudes of the modes composing coiled postures. Scree plots [35] would typically lead to dividing a coiler dataset to k = 6–8 similarly sized clusters. However, we found that generating a larger number of smaller clusters was useful: the centroids of the most populated small clusters resembled postures that were frequently observed in the raw data. Representative examples of cluster centroids and the postures that were reconstructed from them were projected onto the plane of the two leading modes and depicted in Fig 6B.

The dynamics of the amplitudes during continuous periods of coiling could vary between different animals and different types of coils. At the two extremes, the duration of a coil could be spent in a static posture that is not easily released or in a continuous sequence of exaggerated body-bends (also referred to as loopy motion). The amplitudes of the leading modes demonstrate that posterior coils of *egl-30(gf)* and *unc-77/nca-1(gf)* mutants were highly dynamic, such that ventral (positive amplitudes) and dorsal (negative amplitudes) coiling were averaged out while the animal exhibited a continuous succession of non-self-avoiding postures. In contrast, anterior coils of *unc-8(gf)* and *unc-3* mutants were characterized by locking into a static coiled posture (Fig 6C).

More detailed information can be obtained from focusing on specific families of coils. We defined a spool as any posture for which the product of the two leading amplitudes, *a*_1_·*a*_2_, was larger than unity, i.e., anterior and posterior curvatures were sufficiently high and in the same direction. The centroids of the 10 most populated clusters of postures that satisfied this condition spanned the observed range of loops typical of Ω-turns to compact spirals. The shaded areas in the left panel of Fig 6D represent the convex hulls of these centroids for wild-type animals and four coiler mutants. The surrounding postures were reconstructed from 26 of these centroids. The center of the panel, where *a*_1_·*a*_2_ was small, was populated by loops that could typically be observed during Ω-turns. As *a*_1_·*a*_2_ grew larger, we preferentially observed spirals in which the head was at the center in *unc-8(gf)* and *unc-122* mutants. In contrast, we observed a significant fraction of spirals in which the tail was at the center in *egl-30(gf)* and *unc-77/nca-1(gf)* mutants. Taken together with the dynamics of the amplitude, these data suggested that the two pairs of mutants preferentially formed spirals differently. Exaggerating an anterior bend prevented dorsoventral undulations and developed into a static head-centered spiral, perhaps through proprioceptive coupling, i.e., the trigger that compels body regions to bend in the same direction as their anterior neighboring region after a short time delay during forward locomotion [36]. Reversing into an exaggerated posterior bend formed a tail-centered spiral that did not suppress dorsoventral bending and was more rapidly released. Assaying the propagation of body-bends concurrently with coiling (described below) supported this interpretation.

PCA analysis of spools can also be used to assess the severity of a defect. Wild-type animals rarely exhibit postures for which *a*_1_·*a*_2_ >4, but coilers do (Fig 6D, right panel) and this trend was not sensitive to the exact value of the threshold. Projecting the sub group of spools onto its own low-dimensional space could facilitate testing more detailed hypotheses. Intuitively, the resulting three leading principal components corresponded to nearly uniform curvature, tightening of anterior bending, and tightening of posterior bending (S3 Fig). Similarly applying the condition *a*_1_·*a*_2_ < −4 would result in charactering number-8-like coils where anterior and posterior curvature have opposite signs. These results demonstrate that complex posture recognition can be integrated with existing analysis methods for large scale and unbiased studies of severe locomotion defects.

### Initiating directed locomotion promotes coiling in *egl-30(gf)* and *unc-77/nca-1(gf)* mutants

**Fig 7:**
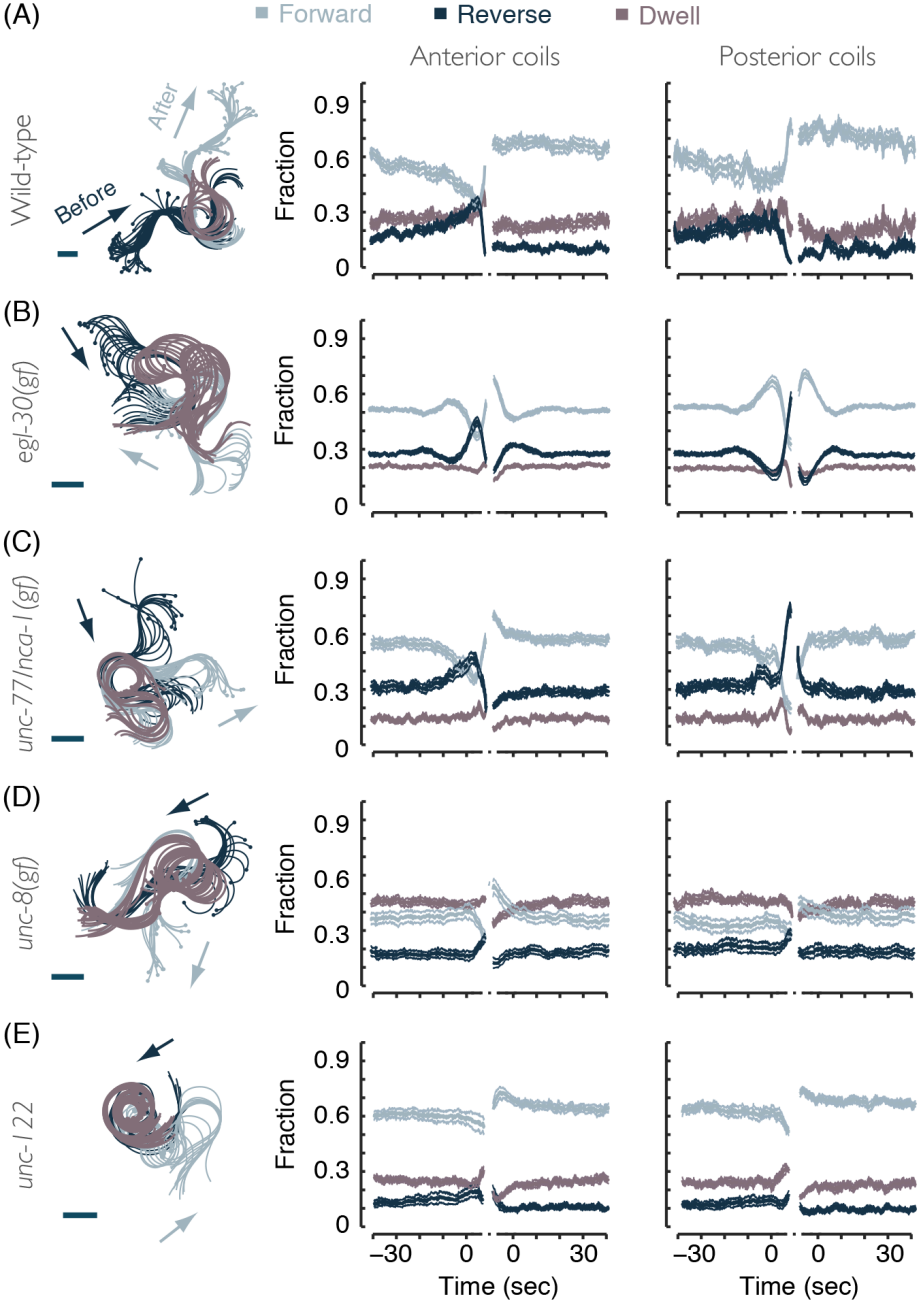
(A) Left: an overlay of postures before, during, and after coiling of a wild-type animal. Arrows depict the direction of locomotion and the scale bar represents 100 μm. Middle: the probabilities of forward locomotion, reversals, and non-directional dwelling before and after a detected period of anterior coiling. Right: locomotion probabilities before and after a period of posterior coiling. In the case of wild-type animals, most coiling events occur during Ω–turns. The horizontal time axis depicts the time leading to and immediately following a continuous period of coiling, i.e., the entry into and exit from a coiling event. The gaps signify that locomotion during the (variable) time of the coiling events themselves is not plotted. (B)-(E) The same as (A) for mutants exhibiting coiling phenotypes. In all panels, 9–12 L4 larvae of each genotype were imaged at 10 frames per second for 2–4 hours. Thin lines depict animal-to-animal variation (mean ± s.e.m).

Is the initiation of directed locomotion particularly favorable for coiling in certain mutant backgrounds? To address this question, we assayed the temporal dynamics of locomotion upon entering and exiting a coiling event (Fig 7). In large part, wild-type coiling resulted from omega turns: acute turns composed of a reversal, an Ω-like posture, and forward locomotion in the new direction [37–41]. As a result, a rise in the propensity to reverse was observed shortly prior to coiling and high levels of forward locomotion were observed immediately following the coil (Fig 7A). In the cases of *egl-30(gf)* and *unc-77/nca-1(gf)* mutants, the signature of a reversal-to-forward switch was detected immediately prior to entering an anterior coil (Fig 7B and 7C, middle panels). Upon posterior coiling, these mutants exhibited the opposite behavioral switch (Fig 7B and 7C, right panels). However, similar trends were not observed in other coiler mutants (Fig 7D and 7E).

**Fig 8:**
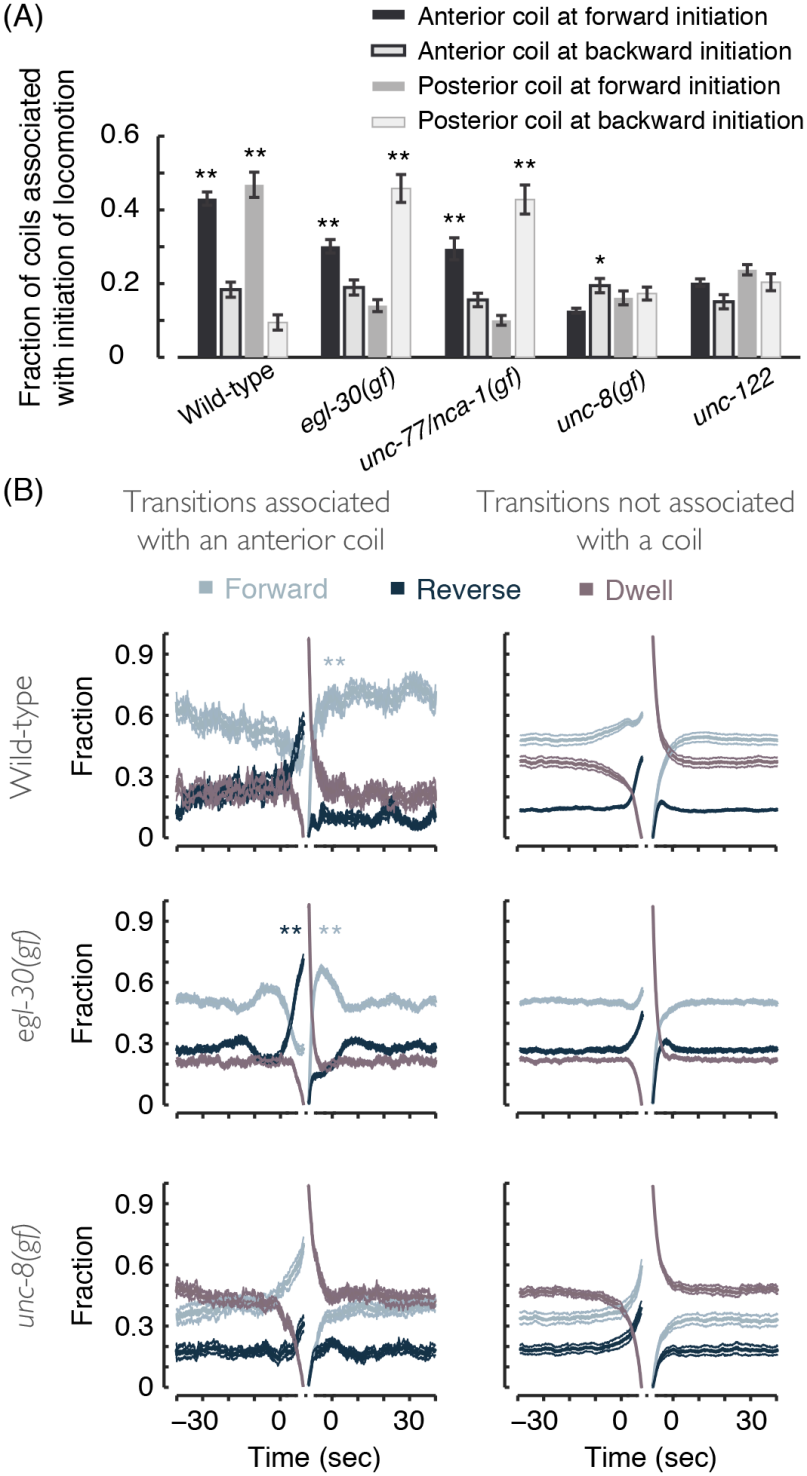
(A) The fraction of coiling events that were detected within 5 sec of the initiation of directed locomotion. Loopy movers exhibit increased probabilities of anterior or posterior coiling upon initiating forward or backward locomotion, respectively. (B) Left: the probabilities of forward locomotion, reversals, and non-directional dwelling before and after a period of dwelling, after the onset of which anterior coiling was detected. Right: the probabilities of locomotion states before and after a period of dwelling, after the onset of which coiling was not detected. The horizontal time axis depicts the time leading to and immediately following a continuous period of dwelling, i.e., the entry into and exit from a dwelling event. The gaps signify that locomotion during the (variable) time of the coiling events themselves is not plotted. In all panels, 9–12 L4 larvae of each genotype were assayed for 2–4 hours. Error bars and thin lines depict animal-to-animal variation (mean ± s.e.m).

As a complementary measure of the association of coiling with locomotion transitions, we measured the fraction of coiling events that occurred within 5 sec from the initiation of directed locomotion. The signature of wild-type Ω-turns could be clearly detected: a large fraction of all coils promptly followed the initiation of forward locomotion after a reversal (Fig 8A). In coiler mutants, an exaggerated posterior body-bend upon a forward-to-reversal switch could increase the likelihood of coiling shortly following the initiation of the reversal. This trend (and the opposite one for anterior coiling) was displayed by *egl-30(gf)* and *unc-77/nca-1(gf)* mutants but not by other coilers (Fig 8A).

Our analysis typically identified brief periods of dwelling during transitions between forward and backward locomotion. Therefore, to visualize selected behavioral trends at the termination and initiation of directed locomotion, we aligned the data at the initiation and termination of short bouts of dwelling. Locomotion was then compared between two sub-categories of the full dataset: events in which, shortly following the onset of dwelling, anterior coiling was identified or no coiling was detected (Fig 8B; additional examples shown in S4 Fig). Coiling upon switching by *egl-30* mutants manifested as an exaggerated reversals peak prior to dwelling and elevated forward propensities following dwelling. Thus, in *egl-30(gf)* and *unc-77/nca-1(gf)* mutants, the dorsoventral bends that initiate directed locomotion may be more likely to exaggerate and result in coiling than those that follow.

### Posterior hyper-bending is asymmetric in *unc-77/nca-1(gf)* mutants

**Fig 9:**
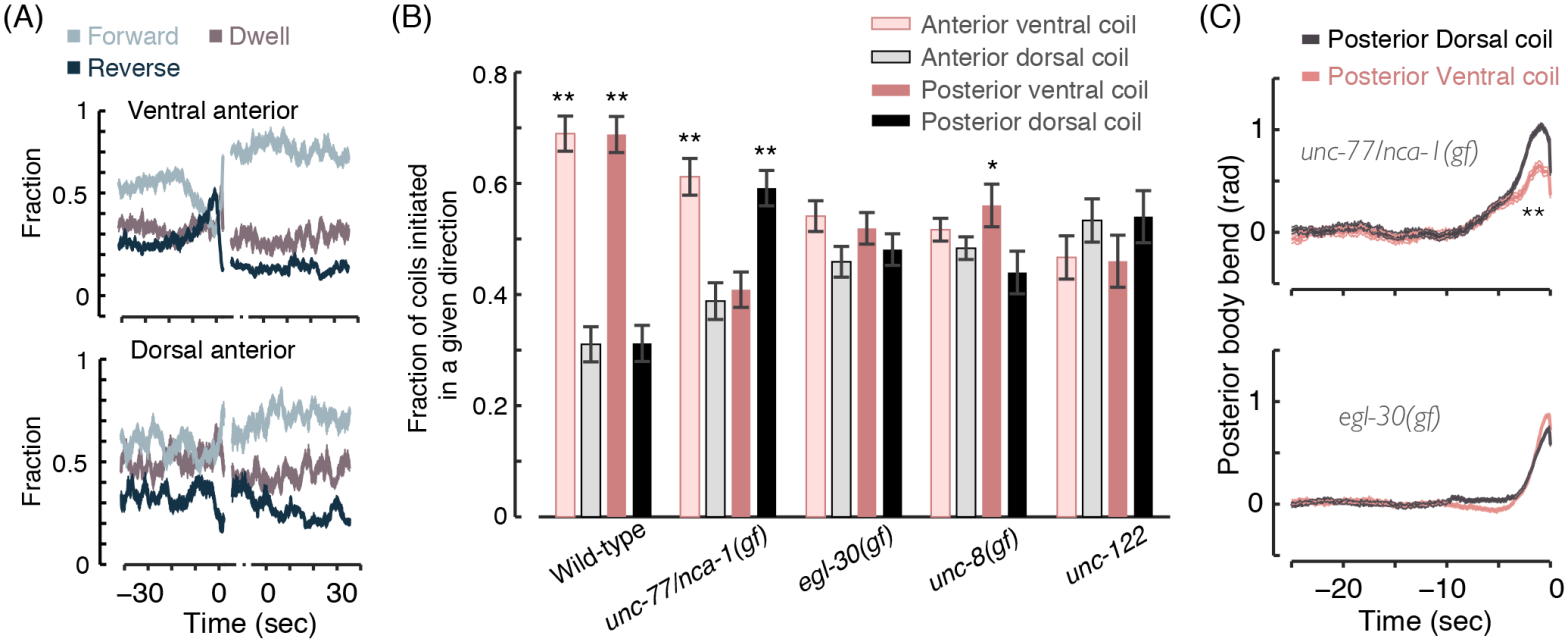
(A) The probabilities of forward locomotion, reversals, and non-directional dwelling of wild-type animals before and after a detected period of anterior coiling. The data was plotted separately for ventral and dorsal coils. The signature of Ω-turns is only apparent in the case of ventral coils. (B) The fraction of the total number of detected coils that were initiated in a given direction. A dorsal preference for posterior coils was only observed in the case of *unc-77/nca-1(gf)* mutants. (C) The posterior relative body angle, i.e., the angle between the most posterior next nearest neighbor intervals out of a total of 20 intervals along the body. Dorsal hyper-bending was more pronounced in *unc-77/nca-1(gf)* (but not *egl-30(gf)* mutants).

Are coiler phenotypes asymmetric with respect to the dorsoventral axis? The deep head bend of an Ω- turn is known to be ventral (Fig 9A) [37–41]. However, ectopic deep bends could potentially arise from the misregulation of bending in either direction. Interestingly, posterior coiling of *unc-77/nca-1(gf)* mutants was more likely when the tail bent dorsally (Fig 9B). The asymmetry in the bending direction of the tail also manifested as higher dorsal (as compared to ventral) posterior curvature in the period leading to a coil (Fig 9C).

The NCA-1 leak channel was recently implicated in persistent motor circuit activity required for sustaining locomotion [42]. Curiously, the gain-of-function of UNC-77/NCA-1 was shown to eliminate some of the spontaneous activity in muscles (miniature postsynaptic currents) [30]. The asymmetric behavior of *unc-77/nca-1(gf)* mutants can lead to hypotheses regarding the structure and function of the backward motor circuit. For instance, given the expression of *unc-77/nca-1* in AVA premotor interneurons, AVA may be capable of asymmetrically activating dorsal and ventral motoneurons. Alternatively, *unc-77/nca-1* may be asymmetrically expressed in motoneurons [43] or not expressed in AS neurons which innervate only dorsal muscles [2,30,44]. In the latter case, AS may play a role in maintaining dorsoventral balance.

We note that the initiation of coiling is not generally restricted to the initiation of directed locomotion. To demonstrate this we examined animals carrying a gain-of-function mutation in the *unc-8* gene, encoding a putative mechanosensory channel [31] or a loss-of-function mutation of *unc-122*, affecting postsynaptic neuromuscular signaling [45]. Neither of these mutants exhibited the signature peaks associated with switching before coiling (Figs 7D, 7E, 8D and 8E). Curiously, *unc-8(gf)* was the only mutant we examined that exhibited significant anterior coiling while reversing, as evident by the unique rise of reversal probability prior to anterior coiling (Fig 7D left and middle panels). These data indicate that the proposed statistical model can be used for testing detailed hypotheses regarding cellular and molecular locomotion mechanisms.

## Discussion

The standard approach for identifying *C*. *elegans* in a digitized image applies simple morphological operations and/or heuristically motivated processing steps [5]. Typically, a background subtraction step is followed by thresholding to obtain a binary image. The largest connected component in the binary image is identified as the animal. Next, morphological closing (dilations followed by erosions) or morphological hole filling is applied and a skeletonization algorithm computes the midline of the body. The head is distinguished from the tail either by manual inspection or by comparing the regions in vicinity of the end points of the midline. Typically, when imaging in “artificial dirt” chambers, the brighter region is associated with the head.

Alternatively, the boundaries of the body in the binary image are determined by subtracting an eroded version of the image (or an equivalent edge detection method). A spline can then be fitted to all boundary points and the end with the higher peak curvature is associated with the tail [10]. If visual inspection is feasible and the duration of the measurement is limited, manual detection of the head and information about the motion of the center of mass can be used to resolve situations where parts of the worm overlap [11–14]. Such approaches are limited in their ability to reliably detect complex (non-self-avoiding) postures based on a single frame.

The approaches described in [11–13] have been applied to non-self-avoiding postures (including some cases of self-crossing midlines) and implemented commercially. They are based upon a geometric model for postures and a motion model for deformations of postures during locomotion. The posture in a given frame is assumed to be a small deformation of the posture in the preceding frame. Given this assumption, complex postures are resolved by tracing them back to simpler ones. These approaches require an initially resolved simple posture that sufficiently resembles the complex one. The simple posture is either provided manually [11] or assumed to be automatically attainable [12,13]. Once such an algorithm loses track of an animal it cannot autonomously recover, but may resume tracking given manual input [11].

These and similar approaches were not specifically designed to address severe phenotypes such as the prolonged continuous periods of coiling exhibited by *egl-30(gf)* mutants. Correspondingly, in published datasets, they were strictly applied to short video sequences in which bouts of coiling were brief. Our formulation of the object recognition problem is qualitatively different: we introduce sparse visual features that enable single-frame detection as opposed to solely relying on the differences in brightness between the imaged animal and its background. In addition to minimizing error propagation and manual intervention, single-frame detection can be parallelized easily and applied efficiently to large datasets.

An enhancement of the standard morphological methods is described in [46], where the skeleton could be determined for omega or spiral shaped postures. In this work, sophisticated heuristics were used to locate and dissect instances of self-touching for certain coiled body configurations. However, this approach is limited to specific postures and cannot be easily generalized. In addition, a laterally coupled snake model was developed for accurate contour detection of coiled animals [47]. When faced with complex postures, this method requires initializations that are close to the correct posture and therefore cannot be used in high-throughput, automated, applications.

Importantly, existing methods do not provide a measure of quality of detection, as they lack a cost function that allows comparison of candidate solutions in a meaningful way. A key advantage of a global generative statistical model is that it is principled: it enables to quantitatively assess the plausibility of the detected posture and can be naturally adapted to different experimental circumstances. An additional advantage of the proposed approach is its scalability. Analyzing a single frame at a time (rather than relying on neighboring frames) is an embarrassingly parallel problem, i.e., one that requires no dependency or communication between the parallel tasks, and eliminates propagation of errors. We implemented our algorithm using open source, freely available tools and libraries that are virtually guaranteed to be available at any research-computing environment. Therefore, our implementation can seamlessly be incorporate in a “big data” workflow for the timely analysis of large volumes of data.

In order to apply our approach to a different species it would be necessary to identify distinct visual features analogous to the edge formations described here. For this work it was sufficient to represent postures as a sequence of instantiation points but more sophisticated representations can be used instead.

To summarize, we presented a computationally efficient method, which correctly detects the posture of *C*. *elegans* in a variety of complex cases where standard morphological operations are inadequate. If higher precision is required, our fine detection method can be extended to a more computationally expensive procedure, e.g., an additional stage of further refinement. The presented analysis of coiler mutants demonstrates the flexibility and usability of this method for generating and testing detailed hypotheses.

## Materials and Methods

### Strains

*C*. *elegans* strains were maintained and grown according to standard protocols [1]. The following strains were used: wild-type strain N2, CG21 *egl-30(tg26); him-5(e1490)*, DR1089 *unc-77(e625)*, CB15 *unc-8(e15)*, CB4870 *unc-122(e2520)*, CB151 *unc-3(e151)*, CB719 *unc-1(e719)*.

### Locomotion assays

Animals were grown at 20°C on standard NGM plates seeded with *E*. *coli* OP50 bacteria. Mid to late L4 individuals were sealed into individual “artificial dirt” chambers filled with an overnight OP50 culture concentrated tenfold and resuspended in NGM medium [48]. Animals were imaged at 10 frames per second at a 4.2x magnification for posture-based analysis using a CCD camera (Prosilica GC2450, Allied Vision Technologies, Stadtroda, Germany). Motion and quiescence were identified using previously described methods [49].

### Analysis of behavior

The proposed algorithm for identifying body features was implemented in Python (performance-critical parts were programmed in Cython) and integrated with our previously described suite of image analysis tools, called PyCelegans [9,49]. In brief, once we identified the body midline, head, and tail in each frame, each midline was divided into 20 equal intervals and the relative angles of all 18 next-nearest neighbor interval pairs (corresponding to the curvature of the body) were calculated. The dynamics of these 18 relative angles were used to identify quiescence and directed locomotion states. The propagation of body bends from anterior to posterior or vice versa was defined as forward or backward locomotion, respectively. Complete lack of motion was defined as quiescence. All other states were defined as dwelling. Although directional propagation of body bends corresponded well to centroid motion, directed locomotion could be scored even if the animal was slipping and the centroid was not propagating in the laboratory frame of reference. Data analysis was performed using custom Matlab scripts (Mathworks Inc., Natick MA). Our source code and documentation are publicly available at https://github.com/david-biron/pycelegans-2.0.

### Statistical and numerical analysis

Data analysis was performed using custom Matlab scripts. For comparisons in summary statistics panels, significance was calculated using a one-way ANOVA test. Post-hoc correction for multiple comparisons was performed using the Bonferroni adjustment. For the purpose of performing principal component analysis (PCA), the posture of the animal was represented using the same 18 relative angles between next-nearest neighbor intervals that were used for the analysis of locomotion. For the purpose of calculating the principal modes, approximately 5,000 anterior coiled frames and 5,000 posterior coiled frames (or spooled, for S3 Fig) were randomly picked from the full dataset of each of the six coiler mutants assayed. Thus, PCA modes were calculated using a total of 60,000 frames. K-means clustering was performed with a redundancy of 5 using k = 50 clusters for Fig 6 and k = 25 for the more restricted set of spools (S3 Fig). Results were not sensitive to an exact choice of the number of clusters, k.

## Supporting Information

S1 Fig The posture of a coiled nematode was similarly detected under different imaging conditions without changing the values of any of the model parameters. (A) The contrast of the image was digitally scaled (top-left = 1.5, top-right = 1.0, bottom-left = 0.75, and bottom-right = 0.5). (B) The top-right image from (A) was blurred using Gaussian filters with standard deviations 2 (left) and 3 (right) pixels.
(TIF)

S2 Fig The distributions of durations (left) and frequencies (right) of continuous periods of coiling of wild-type animals and *egl-30(gf)* mutants. (TIF)

S3 Fig Left four panels: the leading four PCA modes for spools (coiled postures for which *a*_1_·*a*_2_ > 1) in the combined coiler dataset. Right: The variance explained by the modes of the spools in order of their significance. Dashed line represents 95%. Note that in this work postures and eigenworms are represented using angle differences [49] as opposed to angles with a fixed axis [7] (see Methods).
(TIF)

S4 Fig Left: the probabilities of forward locomotion, reversals, and non-directional dwelling before and after a period of dwelling, during the onset of which anterior coiling was detected. Right: the probabilities of locomotion states before and after a period of dwelling, during the onset of which coiling was not detected. The horizontal time axis depicts the time leading to and immediately following a continuous period of dwelling. In all panels, 9–12 L4 larvae of each genotype were assayed for 2–4 hours. Thin lines depict animal-to-animal variation (mean ± s.e.m).
(TIF)

S1 Table A list of the parameters used by the proposed algorithm. The values of the parameters were determined when the code was implemented and remained fixed throughout this work.
(DOCX)

S1 Movie An example of running the statistical algorithm for posture recognition on an *egl-30(gf)* mutant exhibiting loopy motion. Blue circle—head, magenta circle—tail, green line—body midline, red lines—body edges. The dimensions of the observation chamber are 0.95mm x 4mm.
(MOV)

S2 Movie An example of detection of complex postures of an *egl-30(gf)* mutant exhibiting severe coiling. (MOV)
