# Supplementary figures and images for: A Generative Statistical Algorithm for Automatic Detection of Complex Postures
Source: PLoS Comput Biol. 2015 Oct 6;11(10):e1004517. doi: 10.1371/journal.pcbi.1004517 (PMC4595081; doi:10.1371/journal.pcbi.1004517)

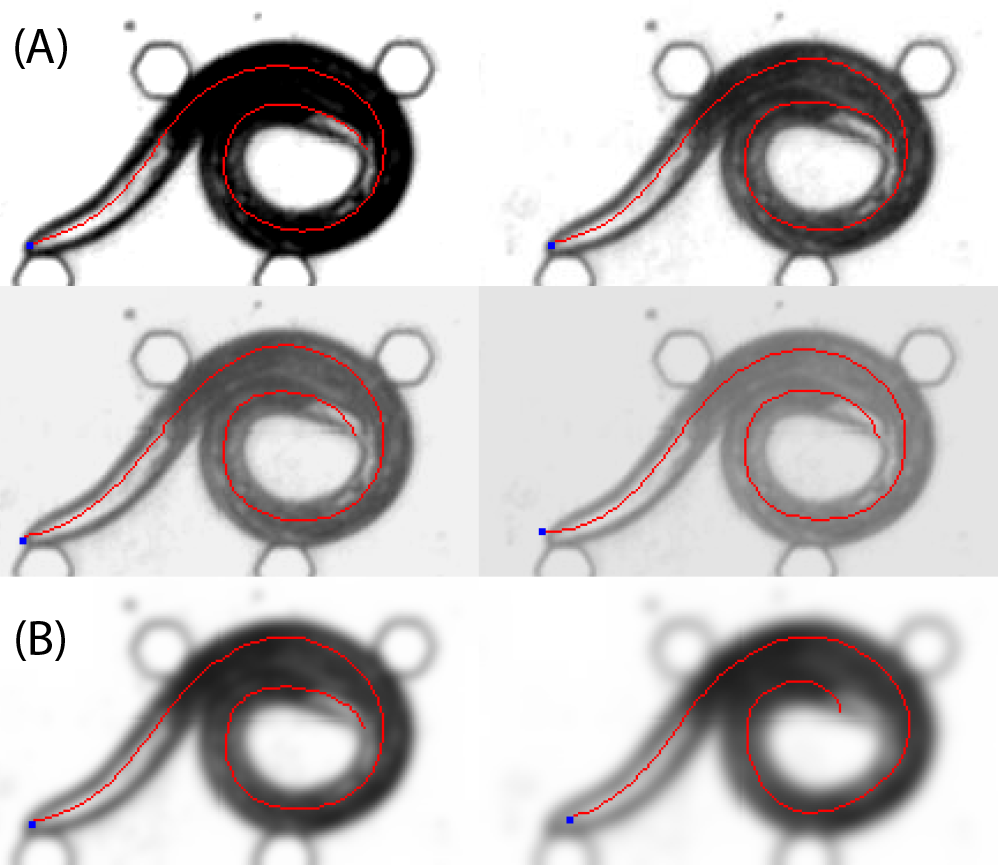

Supplement: S1 Fig — (A) The contrast of the image was digitally scaled (top-left = 1.5, top-right = 1.0, bottom-left = 0.75, and bottom-right = 0.5). (B) The top-right image from (A) was blurred using Gaussian filters with standard deviations 2 (left) and 3 (right) pixels. (TIF) [file pcbi.1004517.s001.tif]

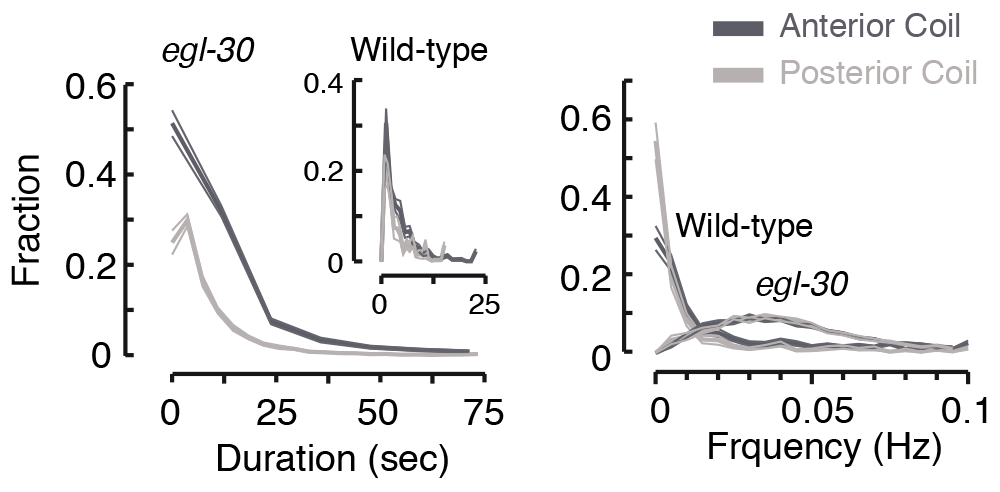

Supplement: S2 Fig — (TIF) [file pcbi.1004517.s002.tif]

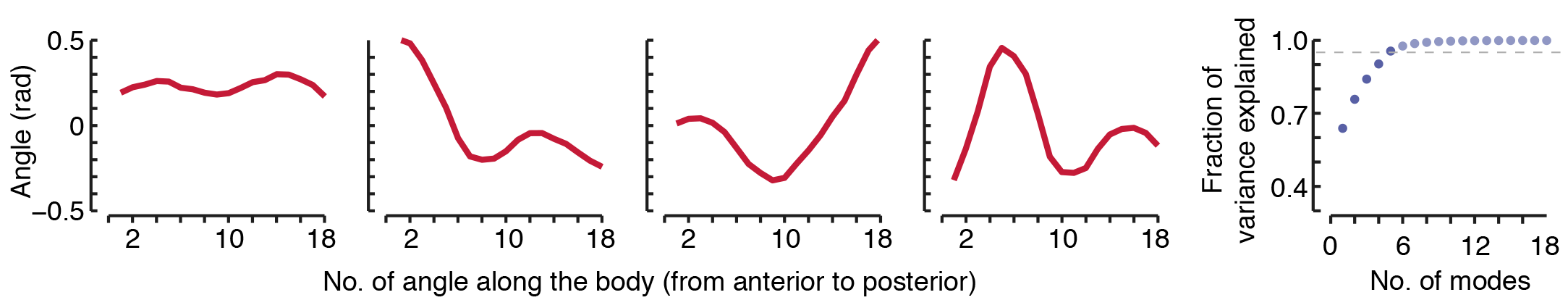

Supplement: S3 Fig — Right: The variance explained by the modes of the spools in order of their significance. Dashed line represents 95%. Note that in this work postures and eigenworms are represented using angle differences [49] as opposed to angles with a fixed axis [7] (see Methods). (TIF) [file pcbi.1004517.s003.tif]

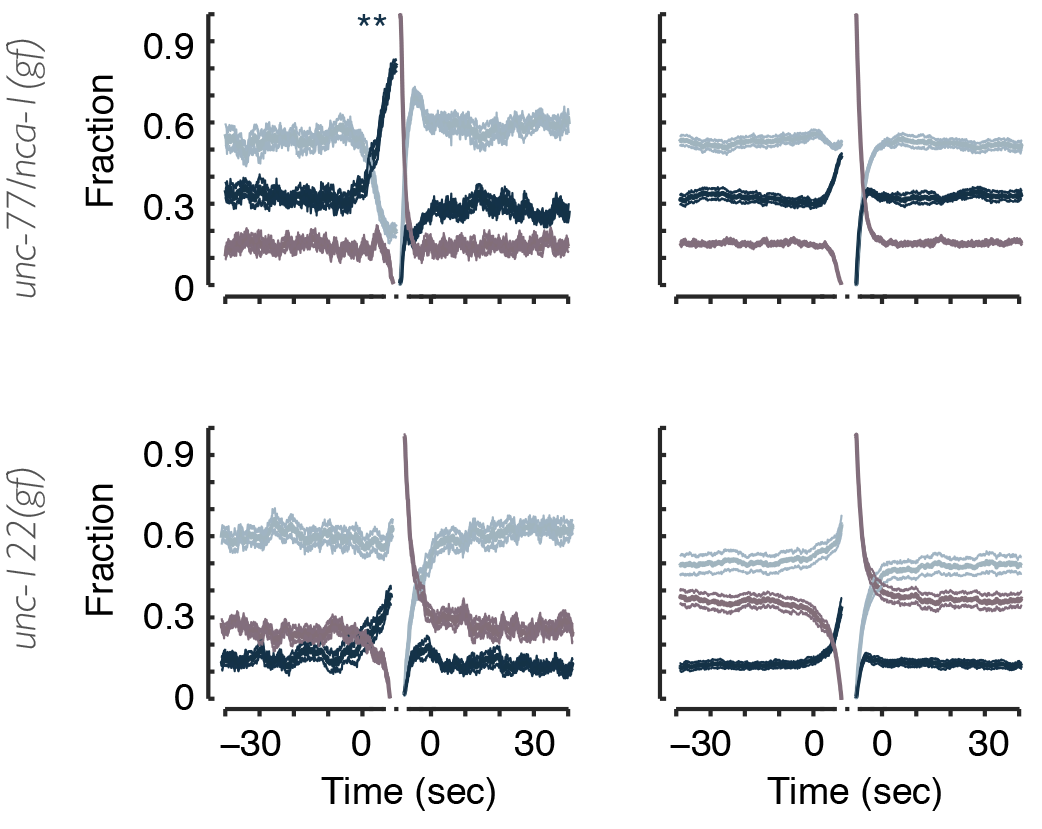

Supplement: S4 Fig — Right: the probabilities of locomotion states before and after a period of dwelling, during the onset of which coiling was not detected. The horizontal time axis depicts the time leading to and immediately following a continuous period of dwelling. In all panels, 9–12 L4 larvae of each genotype were assayed for 2–4 hours. Thin lines depict animal-to-animal variation (mean ± s.e.m). (TIF) [file pcbi.1004517.s004.tif]
