# Supplementary material for: A Generative Statistical Algorithm for Automatic Detection of Complex Postures
Source: PLoS Comput Biol. 2015 Oct 6;11(10):e1004517. doi: 10.1371/journal.pcbi.1004517 (PMC4595081; doi:10.1371/journal.pcbi.1004517)
Supplement: S1 Table — The values of the parameters were determined when the code was implemented and remained fixed throughout this work. (DOCX) [file pcbi.1004517.s005.docx]

| **Parameter name** | **Value used in this work** | **Manner of determination** |
| --- | --- | --- |
| Edge detection threshold | 0.1 (for intensities in [0,1]) | Trial and error |
| Coarse block size | 5 pixels | Quarter worm width |
| Feature detection threshold | 10 | Twice the coarse block size |
| Feature frequencies (low, medium, and high) | 0.3, 0.5, 0.7 | Estimated from small number of images |
| A priori worm length | 70 blocks | Assumed prior knowledge |
| Coarse prior precision | 0.075 | Trial and error |
| Edge frequencies (low and high) | 0.2, 0.7 | Estimated from small number of images |
| Fine prior precision | 1.5 | Trial and error |
